# Predicting Outcome 12 Months after Mild Traumatic Brain Injury in Patients Admitted to a Neurosurgery Service

**DOI:** 10.3389/fneur.2017.00125

**Published:** 2017-04-10

**Authors:** Torgeir Hellstrøm, Tobias Kaufmann, Nada Andelic, Helene L. Soberg, Solrun Sigurdardottir, Eirik Helseth, Ole A. Andreassen, Lars T. Westlye

**Affiliations:** ^1^Department of Physical Medicine and Rehabilitation, Oslo University Hospital, Oslo, Norway; ^2^Faculty of Medicine, Institute of Clinical Medicine, University of Oslo, Oslo, Norway; ^3^KG Jebsen Centre for Psychosis Research/Norwegian Centre for Mental Disorder Research (NORMENT), Division of Mental Health and Addiction, Oslo University Hospital, Oslo, Norway; ^4^Institute of Health and Society, CHARM Research Centre for Habilitation and Rehabilitation Models & Services, Faculty of Medicine, University of Oslo, Oslo, Norway; ^5^Sunnaas Rehabilitation Hospital, Nesoddtangen, Norway; ^6^Department of Neurosurgery, Oslo University Hospital, Oslo, Norway; ^7^Department of Psychology, University of Oslo, Oslo, Norway

**Keywords:** traumatic brain injury, cortical thickness, volumetric analysis, prediction, outcome

## Abstract

**Objective:**

Accurate outcome prediction models for patients with mild traumatic brain injury (MTBI) are key for prognostic assessment and clinical decision-making. Using multivariate machine learning, we tested the unique and added predictive value of (1) magnetic resonance imaging (MRI)-based brain morphometric and volumetric characterization at 4-week postinjury and (2) demographic, preinjury, injury-related, and postinjury variables on 12-month outcomes, including global functioning level, postconcussion symptoms, and mental health in patients with MTBI.

**Methods:**

A prospective, cohort study of patients (*n* = 147) aged 16–65 years with a 12-month follow-up. T1-weighted 3 T MRI data were processed in FreeSurfer, yielding accurate cortical reconstructions for surface-based analyses of cortical thickness, area, and volume, and brain segmentation for subcortical and global brain volumes. The 12-month outcome was defined as a composite score using a principal component analysis including the Glasgow Outcome Scale Extended, Rivermead Postconcussion Questionnaire, and Patient Health Questionnaire-9. Using leave-one-out cross-validation and permutation testing, we tested and compared three prediction models: (1) MRI model, (2) clinical model, and (3) MRI and clinical combined.

**Results:**

We found a strong correlation between observed and predicted outcomes for the clinical model (*r* = 0.55, *p* < 0.001). The MRI model performed at the chance level (*r* = 0.03, *p* = 0.80) and the combined model (*r* = 0.45, *p* < 0.002) were slightly weaker than the clinical model. Univariate correlation analyses revealed the strongest association with outcome for postinjury factors of posttraumatic stress (Posttraumatic Symptom Scale-10, *r* = 0.61), psychological distress (Hospital Anxiety and Depression Scale, *r* = 0.52), and widespread pain (*r* = 0.43) assessed at 8 weeks.

**Conclusion:**

We found no added predictive value of MRI-based measures of brain cortical morphometry and subcortical volumes over and above demographic and clinical features.

## Introduction

Traumatic brain injury (TBI) ranges from severe to mild injuries with approximately 85% classified as mild TBI (MTBI) (1). Most patients with MTBI have a favorable outcome (2), but 5–15% must learn to live with persistent physical, emotional, and cognitive deficits (3). These complaints are known as postconcussion symptoms that may impact long-term postinjury social functioning and work participation (4). Identifying patients at increased risk of poor outcome is essential to aid prognostics and optimize treatment. The advancement of magnetic resonance imaging (MRI) techniques could provide opportunities as prognostic tool in this respect. In a review, Studerus-Germann et al. found that several MRI-based brain imaging modalities, including diffusion tensor imaging (DTI), susceptibility weighted imaging, magnetic resonance spectroscopy, and functional MRI, seem to have adequate sensitivity and specificity as predictive diagnostic tools according to the current literature (5). However, the numbers of studies are small. Blood-based biomarkers for brain damage have long been evaluated as potential prognostic measures in MTBI, but none have emerged thus far as a means of identifying those cases of MTBI with evolving brain damage leading to long-term dysfunction at an early stage (6).

Several factors may increase risk of poor outcome after MTBI. In a systematic review, Silverberg et al. (7) found that potential contributors include preinjury factors (demographic variables including female gender (8), advanced age (8), being single, lower education, poor preinjury health, preinjury functioning, and low level of resilience), injury-related factors (mechanism of injury, severity of MTBI in terms of longer posttraumatic amnesia (PTA) duration, lower Glasgow Coma Scale (GCS) score, and loss of consciousness (LOC), and alcohol intoxication), and postinjury coexistence of pain, posttraumatic stress, anxiety and depression, and negative expectations (7).

Preinjury psychiatric problems (9, 10), premorbid or comorbid physical dysfunction (9), and associated injuries (9, 11) have all been associated with less favorable outcome after MTBI. In a recent Transforming Research and Clinical Knowledge in TBI (TRACK-TBI) pilot study, the strongest predictors of lower functional outcome measured by the Glasgow Outcome Scale Extended (GOSE) at both 3 and 6 months were the demographic and preinjury factors, older age, a history of psychiatric conditions, and lower education (12). The influence of psychological resilience and mood status in conjunction with MTBI remains relatively unexplored. McCauley et al. (13) suggested that preinjury depressed mood and low level of resilience contribute to the severity of postinjury postconcussion symptoms. Low level of resilience has been associated with poor self-reported outcome (14). Also, moderate to severe depressive symptoms in the month prior to injury represent a risk factor for poor behavioral, cognitive, and mental health-related quality-of-life outcomes at 3 months after MTBI (15). Concurrent anxiety, depression, posttraumatic stress (16, 17), and pain (18–20) may contribute to symptoms, and depression and posttraumatic stress have been associated with decreased functional outcome measured with the GOSE (21).

Multiple injury-related factors have been associated with persistent symptoms following MTBI, but findings have been inconsistent (2). The predictive value of GCS has been found to be modest (9, 22). PTA duration greater than 7 days was found to be predictive of residual moderate disability at 6 months postinjury in patients with complicated MTBI (i.e., intracranial injuries) (23). Adding to the heterogeneity, imaging studies have demonstrated that brain pathology, as indicated by CT, is not a strong predictor of outcome with regard to symptoms or global function according to the GOSE in MTBI (11, 24–26). MRI is more sensitive than CT (27), but studies aiming to predict outcome using MRI measures have provided inconsistent results. CT and conventional MRI findings were not predictive of neurocognitive functioning at 1 or 12 months after injury, nor was functional outcome 1 year after injury (25). However, brain contusions and axonal injury within 16 days after injury were stronger predictors for poor outcome in MTBI than demographic factors (28).

In a review of studies of MRI brain volumetry in patients with TBI, the amount of atrophy correlated significantly with important clinical variables, such as LOC, duration of coma, and duration of PTA, and the rate of atrophy was associated with worse long-term functional outcomes after injury (29). Da Costa et al. (30) found significant decrease in gray matter volume between 2 and 6 months after MTBI and a positive correlation between gray matter and SCAT2, a questionnaire assessing current symptoms, with tasks probing cognitive and balance abilities.

However, as a result of the inconsistent findings, the clinical and functional significance of MRI findings is unclear, and there is a need for studies with well-defined, thoroughly characterized cohorts utilizing advanced and automated structural neuroimaging to assess its prognostic value. Understanding how postinjury structural properties of the brain relate to long-term function in MTBI patients is important for developing better predictions of outcomes in clinical settings.

Thus, the main aim of this study was to test the unique and added predictive value of MRI-based brain morphometric and volumetric characterization at 4 weeks postinjury and demographic and clinical data at 12 month-outcome, covering global functioning level, postconcussion symptoms, and mental health. To the best of our knowledge, this is the first attempt to use morphometric and volumetric MRI as features in multivariate prediction models for clinical outcomes in individuals with MTBI.

Based on the literature reviewed above and the notion that interindividual variability in brain structure is sensitive to clinically relevant functions, we hypothesized that MRI-based brain morphometry and volumetry at 4 weeks postinjury would show predictive value for outcomes at 12 months postinjury beyond what can be obtained using clinical and demographic data alone.

## Materials and Methods

### Subjects

Patients were included in a prospective cohort study comprising patients with acute MTBI admitted to Oslo University Hospital during a period from September 2011 to September 2013. The study was approved by the Norwegian Regional Committee for Medical Research Ethics (REC) (2010/1899) and all methods were carried out in accordance with the relevant guidelines and regulations of REC. All participants provided written informed consent.

Mild traumatic brain injury classification criteria vary across studies. Here, MTBI was defined using the criteria from the American Congress of Rehabilitation Medicine (31) and included patients aged 16–65 years with recent (<24 h) history of trauma to the head (hospitalization with ICD-10 diagnosis S06.0–S06.9), resulting in LOC < 30 min, PTA < 24 h, and GCS between 13 and 15. The GCS was registered within the first 24 h following injury, and the lowest GCS score within the first 24 h is reported.

Exclusion criteria were severe mental illness (e.g., major depressive disorder, schizophrenia, or bipolar disorder diagnosed by a psychiatrist or clinical psychologist), progressive neurologic disease, previous ICD-10 diagnosis of substance dependence, contraindications for MRI (including pregnancy and claustrophobia), or lack of Norwegian language skills.

**Figure 1:**
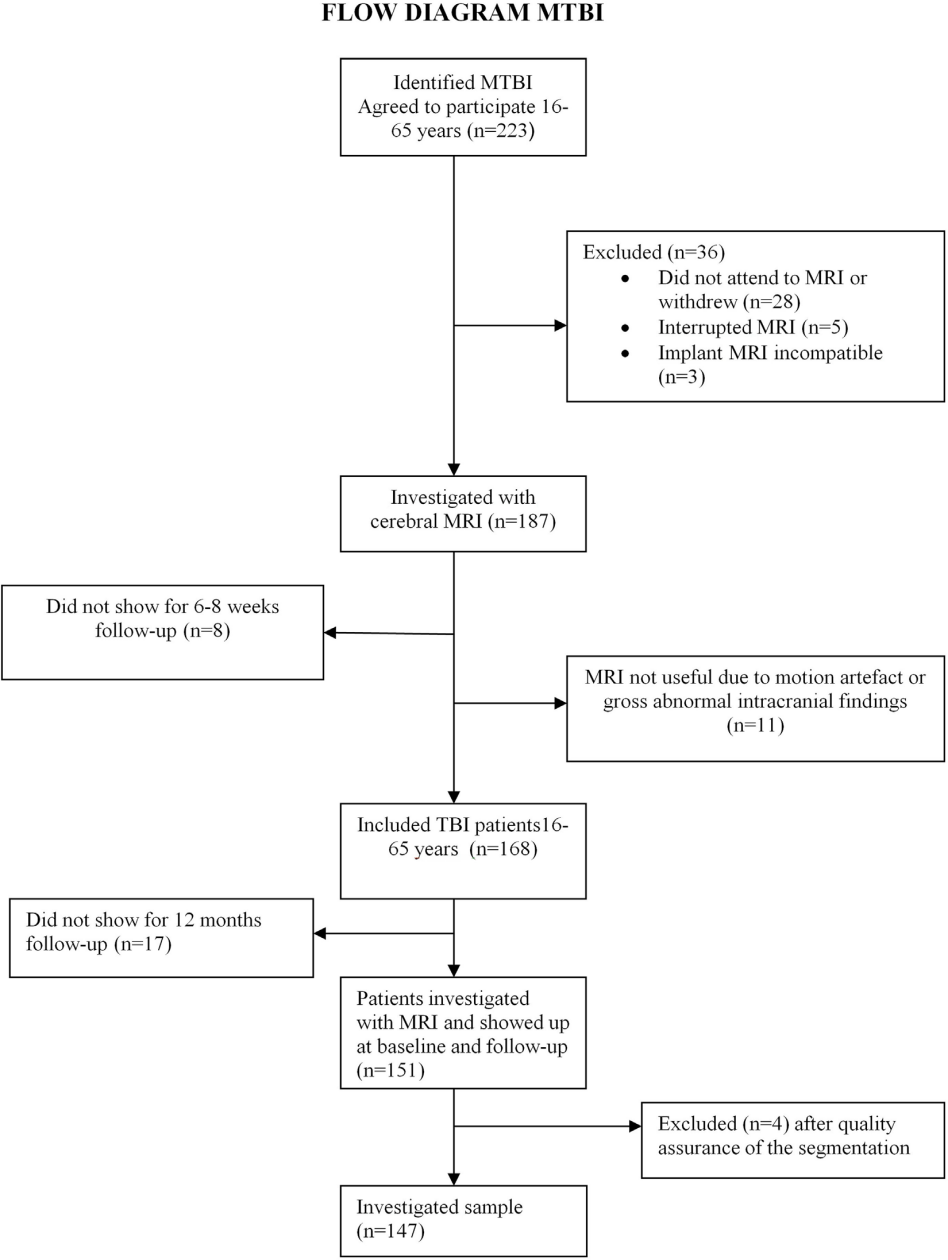
**Flow diagram of mild traumatic brain injury (MTBI)**.

Of the 223 patients who fulfilled the inclusion criteria, 36 were excluded, 28 did not attend to MRI or withdrew, five interrupted MRI, and three had an MRI-incompatible implant. Eight patients did not show up for 8-week follow-up. MRI data sets for 11 patients were discarded due to motion artifact or gross abnormal intracranial findings, which severely interfered with the automated reconstruction in FreeSurfer. Seventeen patients did not show up for 12-month follow-up, and four patients were excluded after quality assurance of the segmentation, resulting in a final sample size of 147 patients (Figure 1).

### MRI Data Acquisition and Analysis

Magnetic resonance imaging was performed using a 3 T whole-body MRI system (Signa HDxt, GE Medical Systems, Milwaukee, WI, USA) with two different head coils: the Head/Neck/Spine coil and the 8HRBRAIN. The protocol included a 3D Fast Spoiled Gradient Echo T1-weighted sequence used for morphometric assessments (repetition time in millisecond/echo time in millisecond/inversion time in millisecond, 7.8/2.96/450; flip angle, 12°; and spatial resolution, 1 × 1 × 1.2 mm). Acquisition parameters were optimized for increased gray/white matter contrast. Additionally, a T2-weighted sequence and a T2 Susceptibility-Weighted Angiography sequence were performed to depict hemorrhagic or other lesions. There was no major scanner upgrade in the study period. All patient MRI data were evaluated for gross pathologies and lesions by a neuroradiologist.

Cortical reconstruction and volumetric segmentation were performed with FreeSurfer (http://surfer.nmr.mgh.harvard.edu/). In short, this processing includes removal of non-brain tissue (32), automated Talairach transformation, segmentation of the subcortical white matter and deep gray matter volumetric structures (33), intensity normalization, tessellation of the gray/white matter boundary, topology correction, and surface deformation (34, 35) to produce representations of cortical thickness. These representations are calculated as the closest distance from the gray/white boundary to the gray/CSF boundary at each vertex on the surface. The maps are not restricted to the voxel resolution of the original data and are capable of detecting submillimeter differences between groups. Using an automated labeling system based on the Desikan–Killiany atlas (36, 37), the cortex was divided into 33 gyral-based regions in each hemisphere. We computed average estimated cortical thickness, surface area, volume, and subjacent white matter volume for major lobes (frontal, cingulate, occipital, temporal, parietal, and insula; https://surfer.nmr.mgh.harvard.edu/fswiki/CorticalParcellation). We also included the volume of the following structures based on standard FreeSurfer volume segmentation algorithms (33): corpus callosum (posterior, mid posterior, central, mid anterior, and anterior), total brain volume (ventricles excluded), total subcortical gray matter volume, total gray matter volume, total supratentorial volume (ventricles excluded), estimated intracranial volume (eTIV), total brain volume to eTIV ratio, lateral ventricles, inferior lateral ventricles, brain stem, cerebellar white matter, cerebellar cortex, thalamus, caudate, putamen, pallidum, hippocampus, amygdala, accumbens, cortical volume, white matter volume, and choroid plexus. The above described procedures yielded a total of 104 brain imaging measures.

### Demographic and Clinical Measures

Selection of preinjury, injury-related, and postinjury predictors was based on prior knowledge from the literature and clinical experience. Clinical data were obtained from the medical records. Postinjury factors were assessed from self-reports obtained at outpatient clinical appointment 8 weeks after injury.

#### Preinjury Factors

Preinjury factors included age in years, gender, marital status (married/partnership/live with and unmarried/widowed/divorced), education (0–12/>12 years), preinjury employment status (yes/no) and in addition preinjury anxiety and depression (yes/no), and resilience measured retrospectively. The Resilience Scale for Adults (RSA) is a 33-item self-report scale measuring resilience protective factors among adults (38, 39). The scale covers six subscales: perception of self, perception of future, social competence, family cohesion, social resources, and structured style as well as a total score. We used both the subscales and the total score in the analyses. This study used a 5-point scale (range 33–165).

#### Injury-Related Factors

The GCS score (40) utilizes the injured persons best eye-opening, verbal, and motor responses to assess the conscious state, with a total score between 3 (showing no response) and 15 (alert and well orientated).

Duration of PTA was assessed in the emergency department (ED) and was classified into no amnesia, less than 1 h, and between 1 and 24 h [Hellstrøm et al. (41), in press].

The presence and duration of LOC were also based on the medical record and classified into no LOC, less than 5 min or 5 min or more [Hellstrøm et al. (41), in press]. The causes of injury were extracted from the medical record and classified as traffic accidents, falls, violence, and others.

Alcohol use was assessed (yes/no) through blood alcohol concentration or clinical examination at the time of hospital admission.

#### Postinjury Factors

##### Pain

Painful areas were recorded using a pain drawing, counting the number of areas (maximum 10 areas) according to Kuorinka et al. (42), including the head. Higher scores indicated widespread pain.

##### Expectation of a Favorable Outcome

In the questionnaire at 8 weeks postinjury, an expectation of a favorable outcome was classified as yes, recovered, no, or do not know the outcome (43).

##### Anxiety and Depression

The Hospital Anxiety and Depression Scale (HADS) (44) is a validated self-assessment scale of anxiety and depression symptoms for the last 7 days, with 14 questions graded on a 4-point Likert scale (0–3), yielding separate anxiety and depression subscale scores of 0–21. The validity and reliability of the HADS have been established in patients with TBI (45, 46). The total HADS score (range 0–42) was used in the analyses.

##### Posttraumatic Stress Symptoms

The Posttraumatic Symptom Scale-10 (PTSS-10) is a self-report rating scale measuring the intensity of posttraumatic stress symptoms (intrusion, avoidance, and hyperalertness). A total symptom severity score (range 10–70) is obtained by summing scores from the 10 items from 1 (never) to 7 (always). It has shown satisfactory psychometric properties with a high internal consistency for the diagnosis of posttraumatic stress syndrome (PTSD) (47, 48). Because the PTSS-10 does not give a formal PTSD diagnosis, we used the term PTSS.

### Outcome Assessment

The dependent variable in this study was determined by combining data from GOSE, Rivermead Postconcussion Symptoms Questionnaire (RPQ), and Patients Health Questionnaire-9 (PHQ-9) obtained during the visit to the outpatient clinic 12 months postinjury into a composite score. We used principal component analysis (PCA) to compute an outcome score reflecting the common variance across GOSE, RPQ, and PHQ-9 (details are given below).

Glasgow Outcome Scale Extended is a global assessment of a functioning tool for the areas of independence, work, social, and leisure activities, and participation in social life (49) recommended as the main outcome measurement in TBI studies in the Common Data Elements (50). It is an 8-point ordinal scale divided into upper and lower levels of good recovery (7, 8), moderate disability (5, 6), severe disability (3, 4), vegetative state (2), and death (1).

Rivermead Postconcussion Symptoms Questionnaire consists of 16 items, which represent the most frequently reported symptoms after MTBI. This instrument covers the cognitive (RPQ cognitive), emotional (RPQ emotional), and physical (RPQ somatic) domains and has been shown to be valid for diagnosing postconcussion symptoms (51). The patients are asked to rate the degree to which each item has become more of a problem during the previous 24 h compared to before the TBI. The responses are then rated on a 5-point Likert scale as follows: 0 = not experienced at all; 1 = no more of a problem; 2 = a mild problem; 3 = a moderate problem; and 4 = a severe problem. The RPQ items are then summed to a total score, excluding ratings of 1 (51).

Patients Health Questionnaire-9 is a reliable and valid measure of the severity of depressive symptoms (52). It consists of nine items that reflect typical symptoms of depression. The response choices assess how often each problem has bothered the patient over the preceding 2 weeks and range from 0 to 3 (not at all to every day). Total score ranges of 0–4 indicate no depression, 5–9 indicate mild depression, 10–14 indicate moderate depression, 15–19 indicate moderately severe depression, and 20–27 indicate severe depression.

### Prediction Models and Cross Validation

We attempted to predict the outcome on the *g* factor based on data from preinjury factors, injury-related factors, and postinjury factors. We formed three prediction models: (A) a brain imaging data model, (B), a clinical data model, and (C) an imaging and clinical data model.

The imaging model included the 104 brain imaging measures, assessed 4 weeks postinjury. The clinical model included the 14 preinjury factors (age, gender, education, marital status, preinjury depression, preinjury anxiety, preinjury work status, and seven resilience factor scores), five injury-related factors (mechanism of injury, GCS on admission, PTA, LOC, and alcohol influence), and four postinjury factors (PTSS-10 total score, HAD total score, expectation of a favorable outcome, and pain), all assessed on admission to the hospital or on outpatient clinical appointment at 8 weeks postinjury. Model C combined all features from both models.

We performed a total of 127 univariate associations (104 imaging features + 23 clinical variables) and corrected for multiple comparison using the false discovery rate (FDR) (53). A support vector regression model was built using a Gaussian kernel with heuristic, automated kernel scaling, and z-standardization applied to the data. Each model was validated using leave-one-out cross-validation. To compare predictive value of the models, we assessed their mean squared error (MSE) as well as the correlation between predicted outcome and the (true) outcome *g* factor. We validated robustness of the model across 10,000 permutations, each randomly permuting the predictor variable.

### Statistical Analysis

Descriptive statistical analyses were performed using SPSS for Windows, version 22 (SPSS Inc., Chicago, IL, USA). Statistical significance was reported at the 0.05 level. PCA, machine learning-based predictions, and corresponding permutation tests were performed using MATLAB 2015b (MathWorks, Inc.). Preinjury, injury-related, and postinjury data are presented as percentage, mean and SD, or median. Next, we performed a PCA on the scores of the RPQ emotional score, RPQ somatic score, RPQ cognitive score, total sum of GOSE, and total score of PHQ-9 and used an alternating least squares algorithm to impute missing data in the PCA framework, whereas missing data were not imputed in the descriptive statistics. Pearson correlation between the composite outcome score and RPQ_emotional_ (*r* = 0.86), RPQ_somatic_ (*r* = 0.86), RPQ_cognitive_ (*r* = 0.90), GOSE_total_ (*r* = −0.78), and PHQ_total_ (*r* = 0.88) indicated that all outcome metrics contributed substantially to the composite score. The subtle variations in the above correlations are likely attributable to varying specificity of the different measures, yet PCA captured the shared variance between all metrics, which was extremely high (73.3% variance explained by the first factor). The outcome composite score did not correlate with age (*r* = −0.088). We then used the resulting factor (*g* factor) explaining the highest proportion of variance across all features as a summary score reflecting functional and symptomatic outcomes. All five variables were equally well represented in this factor (coefficients; RPQ emotional 0.45, RPQ somatic 0.45, RPQ cognitive 0.47, GOSE total 0.41, and PHQ total 0.46). The score explained 73% of the total variance in the five scales, thus rendering it a robust indicator of outcome. A high *g* factor reflected poor outcome.

## Results

### Demographic and Injury-Related Variables

Demographics and injury characteristics are summarized in Table 1. Participants (*n* = 147) were predominately male (63%) with a median age of 40. The leading cause of trauma was traffic injuries (43%). Most patients had LOC less than 5 min (58%) and PTA less than 1 h (71%), LOC and PTA missing for 29 patients.

**Table 1:** **Demographics and injury-related variables of mild traumatic brain injuries (MTBIs) at 8 weeks postinjury**.

Variables	MTBI at baseline (*n* = 147)
Age (years) Mean (SD)	40.0 (13.8)
Gender (*n*, %) – Male – Female	92 (63) 55 (37)
Marital status – Married/partnership/live with – Unmarried/widowed/divorced	94 (64) 53 (36)
Education (*n*, %) – 0–12 years – >12 years	68 (46) 79 (54)
Employment (*n*, %) – Yes – No	123 (84) 24 (16)
Preinjury anxiety – Yes – No	12 (8) 135 (92)
Preinjury depression – Yes – No	13 (9) 134 (91)
Mechanism of injury (*n*, %) – Traffic accidents – Falls – Violence – Others	64 (43) 54 (37) 16 (11) 13 (9)
Glasgow Coma Scale score (*n*, %) – 15 – 14 – 13	107 (73) 34 (23) 6 (4)
Isolated traumatic brain injury (*n*, %) – Yes – No	88 (60) 59 (40)
Loss of consciousness (*n*, %) – Yes (<5 min) – Yes (≥5 min) – No – Unknown	86 (58) 4 (3) 28 (19) 29 (20)
Posttraumatic amnesia (*n*, %) – No amnesia – <1 h – >1 < 24 h – Unknown	13 (9) 104 (71) 1 (1) 29 (19)
Alcohol intoxication admission – Yes – No	44 (30) 103 (70)
Length of acute hospital stay (days) Mean (SD)	2.4 (2.5)
Time to magnetic resonance imaging scan (days)Mean (SD)	36.7 (20)

CT scan at admission was negative in 87 patients, and for five patients, we did not have a CT scan, of which four had a negative and one a positive MRI scan. Twelve patients with a negative CT scan (14%) displayed injury-related findings on MRI (six diffuse axonal injuries, 10 cerebral contusions, and two subdural hemorrhages) and 40% had extracranial injuries. MRI was performed at a median time of 37 days postinjury.

### Self-Reported Symptoms at 8 Weeks

As seen in Table 2, most patients had an expectation of favorable outcome (71%). The mean number of pain areas demarcated was 1.84 (SD 1.7). The mean total HADS score was 7.96 (SD 6.7), and the mean total PTSS-10 score was 21.7 (12.3), indicating low emotional distress and posttraumatic stress symptoms (Table 2). Resilience was missing for five patients, and the distribution of the domains is described in Table 2.

**Table 2:** **Self-reported predictors of MTBIs at 8 weeks postinjury**.

Variables	MTBI (*n* = 147)
RSA total
Mean (SD)	112.5 (18.5)
RSA; perception of self
Mean (SD)	3.28 (0.86)
RSA; perception of future
Mean (SD)	3.27 (0.92)
RSA; social competence
Mean (SD)	3.25 (0.76)
RSA; family cohesion
Mean (SD)	3.49 (0.71)
RSA; social resources
Mean (SD)	3.83 (0.57)
RSA; structured style
Mean (SD)	3.13 (0.78)
Posttraumatic Symptom Scale-10 total
Mean (SD)	21.7 (12.3)
HADS total
Mean (SD)	7.96 (6.7)
Expectation of favorable outcome – Yes – Recovered – No – Do not know	104 (71) 30 (20) 2 (1) 11 (8)
Pain draft
Mean (SD)	1.84 (1.7)

*MTBI, mild traumatic brain injury; RSA, Resilience Scale for Adults, missing 5; HADS, Hospital Anxiety and Depression Scale*.

### Functional and Symptomatic Outcome 12 Months Postinjury

Table 3 presents the mean and SD of the functional (GOSE) and symptomatic outcome (RPQ and PHQ-9) 12 months after injury. The results indicate good functional outcome [GOSE mean 7 (SD 1)] and relatively low symptom burden [RPQ mean 13.12 (SD 14.0), PHQ-9 mean 6.45 (SD 5.45)] at 12 months postinjury.

**Table 3:** **Self-reported outcome measures of MTBIs at 12 months postinjury**.

Variables	MTBI (*n* = 147)
RPQ total
Mean (SD)	13.12 (14.0)
RPQ somatic
Mean (SD)	6.39 (7.2)
RPQ emotional
Mean (SD)	3.40 (4.3)
RPQ cognitive
Mean (SD)	3.33 (4.0)
GOSE
Mean (SD)	7 (1)
PHQ-9
Mean (SD)	6.45 (5.45)

*MTBI, mild traumatic brain injury; RPQ, Rivermead Postconcussion Symptoms Questionnaire; GOSE, Glasgow Outcome Scale Extended; PHQ-9, Patient Health Questionnaire-9*.

### Outcome Prediction

**Figure 2:**
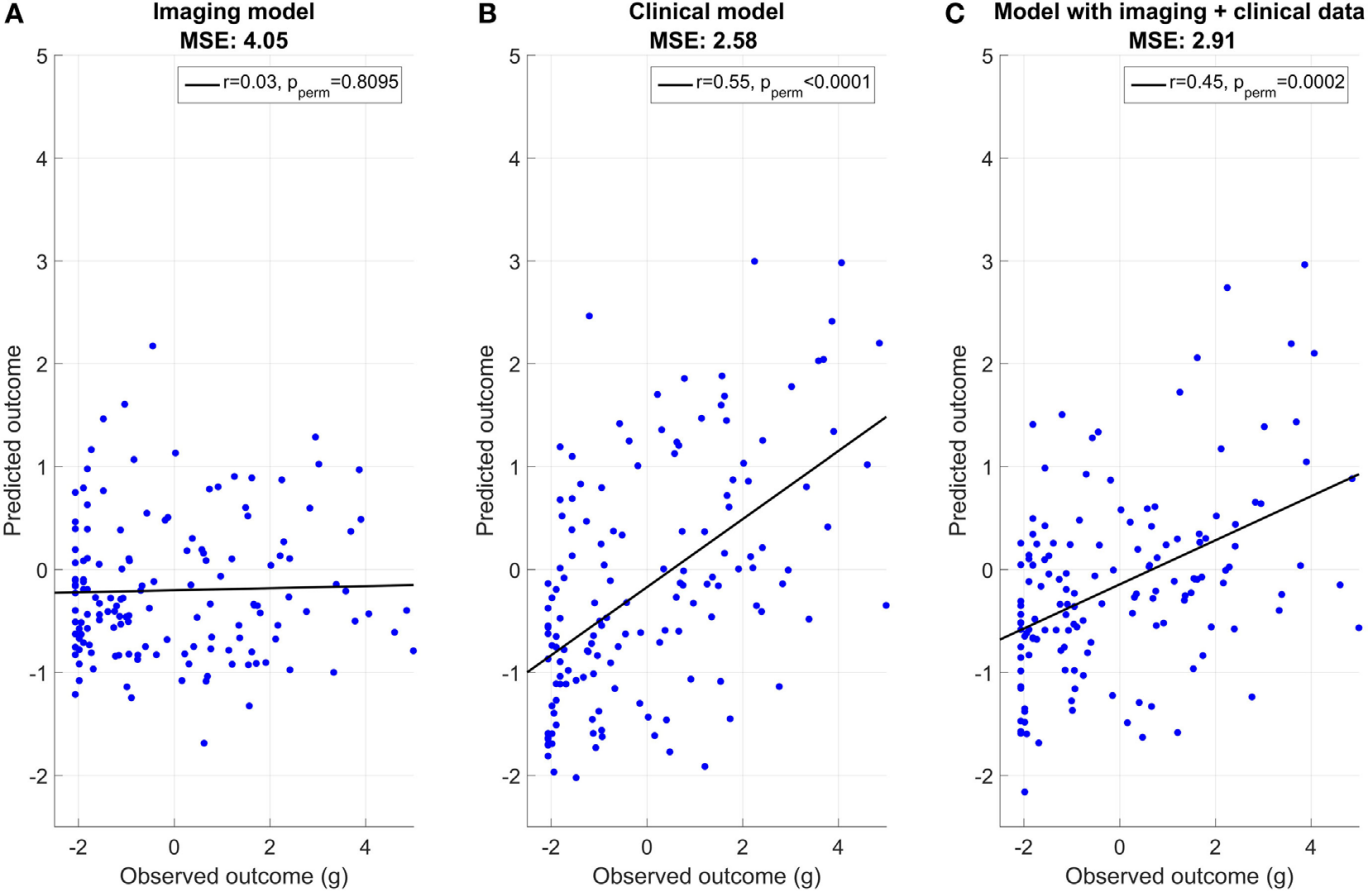
**Association between predicted and observed outcome. (A)** Imaging model mean squared error (MSE): 4.05, **(B)** clinical model MSE: 2.58, and **(C)** model with imaging + clinical data MSE: 2.91.

Figure 2 shows the association between predicted and observed outcomes. With an MSE of 2.58 and a correlation between observed and predicted outcomes (*r* = 0.55), the clinical model (model B) yielded significantly better prediction accuracy than the imaging model (MSE 4.05, *r* = 0.03). Predictions in model A (MRI data only) did not perform significantly above chance (*p* = 0.80, *p* value obtained across 10,000 permutations), whereas those in model B (clinical data only) were well above chance (*p* < 0.001). The prediction accuracy of the imaging model with age incorporated as a feature did not improve (MSE: 4.06). Adding MRI and clinical data in one model (model C) did not increase performance (model 3, MSE 2.91, *r* = 0.45, *p* < 0.002) compared to the clinical model.

Univariate correlations with outcome revealed seven FDR significant correlations (*p* ≤ 0.0017) including six clinical [three RSA (RSA perception of self, RSA perception of future, and RSA total), PTSS, HADS, and pain] and one imaging feature (the volume of the left choroid plexus).

Strongest association was found with posttraumatic stress (PTSS-10, *r* = 0.61), psychological distress (HADS, *r* = 0.52), and pain (*r* = 0.43). Higher resilience was associated with better outcome (RSA perception of self: *r* = −0.34, RSA perception of future: *r* = −0.39, and RSA total: *r* = −0.26). The volume of the left choroid plexus (*r* = −0.26) and the midposterior part of the corpus callosum (*r* = 0.19, not surviving FDR correction) were the MRI features showing the strongest associations with outcome, with a smaller choroid plexus and a larger corpus callosum associated with worse outcomes. Partial correlations accounting for age revealed the same seven significant (FDR-corrected) associations.

## Discussion

Accurate prognostic estimates are critical for clinical decision-making, but the added value of advanced MRI brain morphometric characteristics for prognostic purposes has remained unclear. The main result in this study is that we found no added predictive value of morphometric and volumetric measures obtained 4 weeks postinjury on a continuous outcome measure based on a composite score of GOSE, RPQ, and PHQ-9 at 12 months after injury in patients suffering from MTBI who had been admitted to the hospital within 24 h of injury. Comparing three multivariate prognostic models revealed that the model including demographic and clinical features yielded significantly better prediction than a model including imaging data alone and the merged model. The most robust prognostic factors obtained at 8 weeks postinjury, when considered alongside others in the multivariate models, were posttraumatic stress symptoms (PTSS-10), psychological distress (HADS), and widespread pain.

Magnetic resonance imaging has been shown to be more sensitive than CT in detecting subtle lesions during the acute phase (26, 54). Despite this, neither CT nor MRI neuroradiological findings were found to account for cognitive impairment in the study of Lee et al. (25), suggesting that alternative imaging techniques are needed to provide imaging-based predictors of outcome in MTBI. Here, we assessed the predictive value of a sensitive and automated approach for quantifying morphometric properties of the brain, including cortical thickness and subcortical volumetry. Although the sensitivity of these measures has been demonstrated repeatedly across a range of conditions and disorders (55, 56), the multivariate model including clinical variables outperformed the neuroimaging model for predicting outcome, and adding neuroimaging features to the clinical model did not increase accuracy. The two volumetric measures showing the strongest associations with outcome were the volumes of the lateral ventricle choroid plexus and the midposterior part of the corpus callosum, of which only the first survived corrections for multiple comparisons. A smaller choroid plexus and a larger corpus callosum were associated with worse outcomes.

The choroid plexus is a collection of cells located in the ventricles. In addition to the production of cerebrospinal fluid (CSF), its tasks include filtration and cleaning of the CSF, indicating an important role in maintaining optimal brain function. Interestingly, TBI frequently results in neuroinflammation, which includes the invasion of neutrophils. A review article concluded that functional destabilization of the choroid plexus and ependymal wall in the brain following TBI has marked effects on CSF homeostasis and periventricular neurogenic viability (57). Using a morphometric MRI analysis, a previous study reported larger estimated choroid plexus volume in patients suffering from complex regional pain syndrome (58). Although the current implication of choroid plexus-related processes (e.g., neuroinflammation and CSF/brain maintenance and homeostasis) in the pathophysiology of MTBI is intriguing, the choroid plexus has received relatively little attention in clinical neuroscience, partly because a precise delineation of the structure using MRI data is often difficult. Therefore, the current results need to be interpreted with caution and should be replicated and investigated in independent samples and with other MRI sequences specifically targeting neuroinflammatory mechanisms.

The gray–white matter junction and midline brain structures are particularly vulnerable to diffuse axonal injury, and the corpus callosum and dorsolateral midbrain are frequently involved (59). Studies have found reduced white matter integrity in the corpus callosum (60, 61), which has also been associated with functional outcome using The Functional Independence Measure and Glasgow Outcome Scale, although in severe TBI (62). Importantly, we did not include DTI-based indices of white matter microstructure in the present analysis, and the relationship between DTI-based measures and white matter volume is complex (63). The results from our study need to be verified, but the findings pinpoint the corpus callosum as a potential target for future research.

Mental health including levels of anxiety has been associated with outcome after MTBI in several studies (10, 17, 19, 64). Posttraumatic stress has been implicated as an outcome predictor (9, 19), and both depression and PTSD have been associated with decreased functional outcome measured with GOSE (21). Recently, it was demonstrated that patients reporting ongoing PCS at 12 months of follow-up exhibited a psychological risk factor at 1 month (e.g., depression, possible traumatic stress, and/or low resilience) (65). Indeed, it has been suggested that preinjury depressed mood and low level of resilience are significant contributors to the severity of postinjury anxiety and postconcussion symptoms, even after accounting for effects of other host factors (13). Another study reported that resilience was associated with self-reported outcome after MTBI (14), which is consistent with our current results. These converging findings emphasize the importance of considering emotional well-being after injury, and early mental health interventions may be beneficial for recovery.

A literature review concluded that headache and bodily pain represent inconsistent predictors of outcome (7). Headache is shown to be predictive of outcome measured by RPQ at 3 months postinjury (66), and patients with low levels of pain early after injury had good recovery measured by return to work (9). Acute pain has been significantly associated with postconcussive symptoms at 3 months of follow-up, but the generalizability to the current study is unclear due to the predominance of 40% assaults (20) compared to 11% in this study. High levels of pain have been related to adverse early MTBI outcomes (67), and negative MTBI perceptions were associated with PCS at 6 months (64). In contrast, our multivariate prediction models did not yield a relevant predictive value of patient expectations of favorable outcome, which may be because the instrument does not measure any specific components of patients’ perceptions of their illness and the future.

Preinjury factors including age, gender, education, marital status, preinjury health, and preinjury work status did not contribute uniquely to outcome prediction in our study. A recent review article concluded that gender was not a well-studied prognostic indicator for recovery after MTBI, but small gender differences were found for some outcome variables (68), with worse outcome for female patients. Older age has been shown to be a significant predictor of global functioning at 3 and 6 months after MTBI (12), which is consistent with a recent review concluding that older adults may be more vulnerable to poor outcomes than younger adults (7). Findings in this study indicate no age effects probably because older adults (>65 years) were excluded. The prognostic value of education level in most studies is not related to symptomatic outcome (69), but in some studies it is related to functional outcome (9, 12). Possible explanations of the education not being a predictor in our study could be related to characteristics of the composite outcome measure and that education level is typically relatively homogeneous in Norway. Although earlier studies have reported that married people with a disability have fewer problems (70) and longer life expectancy (71) compared to their unattached counterparts, marital status was not predictive of outcome in this study.

Although preinjury mental health is shown to be a robust prognostic factor in several studies (10, 12, 19, 72), our models did not reveal any relevant predictive value of preinjury anxiety or depression. However, preinjury mental health status was based on self-reports that may be less sensitive and more biased than more objective assessments. Another likely explanation is that our patients had a relatively high functional level preinjury, and thus, residual limitations may influence their ability to regain their former psychological problems. Additionally, we excluded patients with severe mental illness. Being sick-listed before injury as a measure of preinjury functioning has been shown to contribute to postinjury functioning measured as return to work (73). However, in the present study, work status (yes/no) did not contribute uniquely to outcome above the other preinjury predictors.

In accordance with a recent comprehensive literature review (7), traditional injury characteristics such as GCS, LOC, PTA duration, and mechanism of injury did not predict outcome in this study. Another study revealed that extracranial injuries and lower GCS were predictive of poorer functional outcome after mild TBI caused by assault (12). A study combining several measures found no predictive value of GCS, LOC, PTA, and abnormal CT findings for MTBI recovery (9), which is consistent with another study reporting no predictive value of CT pathology (24).

Various outcome measures are used in prognostic analysis for MTBI. The GOSE is often used for global outcome and RPQ for self-reported symptoms. The coarseness of GOSE makes it less sensitive to subtle dysfunction, which is typically observed in patients with MTBI, and may, therefore, not permit sufficient differentiation of outcome in patients with milder injuries (74). GOSE also does not discriminate between physical and mental disabilities. It has been reported that GOSE was uniformly high for MTBI patients at 1 year after injury and may not be sensitive to specific neurocognitive deficits in specific domains as more specialized tests (25). RPQ is sensitive to postconcussion symptoms but is a gross outcome measure and also grades common symptoms that are not specific to MTBI. Because composite outcome measures are recommended (74), we created a composite outcome score using PCA based on global functioning and self-reported cognitive, emotional, somatic, and mental health symptoms (GOSE, RPQ and PHQ-9, respectively).

This study has several limitations which should be considered in interpreting the findings. Our sample size was relatively small for prediction modeling. Additionally, only MTBI patients requiring neurosurgical consultation at the ED and hospitalization were included, which may have caused inclusion bias. Our models may, therefore, have the most value for more severely injured MTBI patients, and generalization of the findings to all MTBI patients should be made with caution. Neuropsychological dysfunctions are usually not seen in sport athletes after 1–3 weeks (75), and Ivins et al. (76) reported that 23% of active duty soldiers sustained TBI after joining the army.

Magnetic resonance imaging was undertaken approximately 4 weeks postinjury, we have no preinjury MRI to compare with postinjury MRI, and acute changes present within 24 h may have disappeared 4 weeks later. More studies are needed to clarify the importance of different time intervals between injury and scanning. The use of potential different prognostic measures like blood-based biomarkers would possibly have strengthened the study, but none have so far been able to identify those cases of MTBI with evolving brain damage leading to long-term dysfunction. Also, the included volumetric estimates do not allow for a detailed characterization of the neurobiological underpinnings. PTA and LOC were unknown for several patients, and we had five missing data for resilience. We have used imputation procedures based on a least squares algorithm to replace missing values with the predicted estimate.

The lack of a control group either without injury or with non-head injury makes it impossible to determine whether the factors associated with MTBI prognosis in our study are specific to MTBI. It is also difficult to compare results across various MTBI prognostic studies due to the heterogeneity in the definition of MTBI, the variety of outcome measures, and the variability in time elapsed for scoring both predictors and outcome. It is important to choose a measure assessing clinically meaningful outcome, and we, therefore, created a factor of GOSE, RPQ, and PHQ-9 for outcome evaluation because it comprises mental, cognitive, physical, and global functioning level of postinjury MTBI. This measure, as in many other MTBI studies, is subjective and requires further validation. The outcome was measured at 12 months of follow-up, which is consistent with previous recommendations (77). Additionally, although we addressed many factors, there are other potentially relevant variables that we did not include, such as early cognitive testing.

Reliable outcome prediction of MTBI remains difficult, despite major progress in brain imaging techniques. There is need for supplementary tests to enable early prediction, both to select appropriate management strategies and to determine the need for prolonged follow-up. Contrary to our hypothesis, MRI morphometry and volumetry did not provide predictive value for outcome at 12 months postinjury over and above the information captured by conventional clinical variables. However, this finding does not exclude brain pathology as a factor at play in patients with poor outcome after MTBI. First, despite the better sensitivity to brain pathology, it could be that the imaging method used in this study may not be sensitive to relevant functional and structural abnormalities, and further studies should include a broader range of MRI modalities, including structural, functional, and neurochemical imaging. There are needs for large cohort studies like TRACK-TBI, The Chronic Effects of Neurotrauma Consortium, and Center TBI, and hopefully these studies will provide answers to some of the questions under study. Second, it is also possible that imaging features may be associated with other aspects of functional outcome not included in this study and which may be affected in MTBI patients (e.g., specific cognitive functions assessed using experimental or neuropsychological tests).

## Conclusion

This study has shown no added predictive value of cortical morphometry and subcortical volumes over and above the information provided in the demographic and clinical features. The current results will need further validation in large longitudinal studies in independent samples using a more comprehensive set of neuroimaging methods that hopefully will clarify the possible role of structural and functional brain abnormalities for long-term outcome after MTBI. The findings, however, support the view that factors other than brain injury deserve attention to minimize long-term complaints after MTBI.

## Author Contributions

TH, NA, HS, OA, SS, and EH took part in design of the study. TH collected data and analyzed the demographic part, LW was in charge of FreeSurfer analysis, and LW and TK were responsible for the prediction model and the description of the model. SS was responsible for the neuropsychological test battery. NA was the main supervisor. All authors discussed the results and implications and commented on the manuscript at all stages.

## Conflict of Interest Statement

The authors declare that the research was conducted in the absence of any commercial or financial relationships that could be construed as a potential conflict of interest.
